# Awareness and intention to use event‐driven and long‐acting injectable pre‐exposure prophylaxis among adolescent and young men who have sex with men and transgender women in Brazil: a cross‐sectional study

**DOI:** 10.1002/jia2.26479

**Published:** 2025-07-02

**Authors:** Laio Magno, Beo Oliveira Leite, Alexandre Grangeiro, Lorenza Dezanet, Fabiane Soares, Inês Dourado

**Affiliations:** ^1^ Departamento de Ciências da Vida Universidade do Estado da Bahia Salvador Brazil; ^2^ Instituto de Saúde Coletiva Universidade Federal da Bahia Salvador Brazil; ^3^ Faculdade de Medicina Universidade de São Paulo São Paulo Brazil; ^4^ Faculdade de Medicina Universidade Federal de Minas Gerais Belo Horizonte Brazil

**Keywords:** Brazil, cabotegravir, HIV prevention, HIV pre‐exposure prophylaxis, sexual and gender minorities, youth health

## Abstract

**Introduction:**

New pre‐exposure prophylaxis (PrEP) options, including event‐driven and long‐acting injectable, may enhance HIV prevention strategies among adolescents and youth. This study examined awareness and intention to use event‐driven and long‐acting injectable PrEP, along with associated factors, among adolescent and young men who have sex with men and transgender women.

**Methods:**

A cross‐sectional study was conducted between December 2020 and February 2022 among men who have sex with men and young transgender women aged 15–20 years, who participated in a daily oral PrEP cohort study in Salvador and São Paulo, Brazil. Binomial logistic regression models analysed factors associated with the intention to use event‐driven and long‐acting injectable PrEP.

**Results:**

A total of 1221 participants were enrolled in the cohort at the time of this analysis, with 597 responding to the survey. Awareness of event‐driven and long‐acting injectable PrEP was reported by 15.3% and 18.0% of participants, respectively. Intention to use event‐driven PrEP was reported by 56.4% of participants, while 81.5% expressed intention to use long‐acting injectable PrEP. Participants with lower and moderate adherence to daily oral PrEP were more likely to intend to use event‐driven PrEP (OR = 1.79; 95% CI: 1.04–3.08), whereas those who reported always or often using condoms in insertive anal sex with steady or casual partners were less likely to intend to use event‐driven PrEP (OR = 0.37; 95% CI: 0.15–0.90). For long‐acting injectable PrEP, participants with middle (OR = 1.93; 95% CI: 1.05–3.53) or low socio‐economic status (OR = 3.13; 95% CI: 1.30–7.51) and those reporting three or more casual partners in the past 3 months (OR = 2.25; 95% CI: 1.30–3.89) were more likely to intend to use long‐acting injectable PrEP. Conversely, participants who had never used daily oral PrEP were less likely to intend to use long‐acting injectable PrEP (OR = 0.31; 95% CI: 0.11–0.92).

**Conclusions:**

Adolescents and young people in Brazil demonstrated a stronger preference for long‐acting injectable over event‐driven PrEP, with sexual behaviour patterns significantly influencing choices. Expanding prevention options may enhance PrEP uptake and adherence, improving HIV prevention strategies among adolescents and young adults.

## 1 INTRODUCTION

The HIV epidemic has increasingly affected young people and adolescents in Latin America [1, 2]. In Brazil, HIV rates continue to rise [3], particularly among men aged 15–24 years, with the incidence rate increasing from 6.4 per 100, 000 in 2000 to 12.8 in 2015, reaching 72.5 per 100, 000 inhabitants with an annual growth rate of 3.7% [4]. Adolescents and young men who have sex with men (AYMSM) and transgender women (AYTGW) are disproportionately affected by the HIV epidemic in Brazil [1, 2, 5, 6]. Additionally, studies have documented declining condom use among youth over the years [6, 7], earlier age of sexual debut [7], and challenges in oral pre‐exposure prophylaxis (PrEP) continuation [8] and adherence [9].

Expanding PrEP options may enhance uptake and adherence among adolescents and youth by allowing individuals to choose the method that best meets their needs. Event‐driven PrEP (ED‐PrEP) [10] may be suitable for those who prefer not to take daily medication or whose sexual activity varies over time [11]. In contrast, long‐acting injectable PrEP (LAI‐PrEP) demonstrated superior efficacy in preventing HIV acquisition among men who have sex with men (MSM) and transgender women (TGW compared to daily oral PrEP [12], making it a promising option for those who either prefer or are unable to adhere to a daily regimen. While ED‐PrEP and LAI‐PrEP with cabotegravir were recommended by the World Health Organization (WHO) in 2019 and 2022, respectively [10, 13], ED‐PrEP was only implemented in the Brazilian National Health System (SUS) in 2023 [14], and LAI‐PrEP with cabotegravir has not yet been introduced.

To date, no data are available on awareness of and preferences for different PrEP modalities among AYMSM and AYTGW in Brazil. Therefore, we analysed the awareness and intention to use ED‐PrEP, a dosing regimen in which two pills are taken 2−24 hours before sexual intercourse, followed by a third pill 24 hours after the initial dose and a fourth pill 48 hours after the initial dose, and LAI‐PrEP, a form of PrEP administered via intramuscular injection that provides HIV protection for 2 months (i.e. cabotegravir). Additionally, we examined factors associated with these preferences among AYMSM and AYTGW participating in a daily oral PrEP cohort study in Brazil.

## 2 METHODS

### 2.1 Study design and population

This is a cross‐sectional study nested within the PrEP15‐19 cohort, the first project in Latin America designed to assess the effectiveness of oral PrEP among AYMSM and AYTGW. This study was conducted at the Salvador and São Paulo sites of the PrEP15‐19 cohort, originally implemented in three Brazilian cities. The inclusion criteria for PrEP15‐19 were self‐identification as AYMSM or AYTGW, being 15−19 years old at the time of enrolment and residing in or regularly engaging in activities within the study cities. The exclusion criterion was the presence of a mental disorder that prevented participation in the study. A specialized psychological and clinical assessment was conducted after enrolment to identify any mental and/or intellectual impairments that could compromise PrEP use. Participants were enrolled in one of two study arms based on their preference: (1) the PrEP arm for individuals who opted to receive daily oral PrEP and (2) the non‐PrEP arm for those who chose alternative HIV prevention methods. Further details about PrEP15‐19 can be found in Dourado et al. [15].

Demand creation strategies for participant recruitment were implemented at each site and included outreach by peer educators in Lesbian, Gay, Bisexual, Transgender, Queer, Intersex and Asexual youth venues; mobilization efforts within non‐governmental organizations, schools and digital platforms such as social media and hook‐up apps [16]; and respondent‐driven sampling (RDS), a peer recruitment method leveraging participants’ social networks. This analysis included adolescents enrolled in the PrEP15‐19 study who resided in Salvador and São Paulo and completed a questionnaire assessing awareness and intention to use ED‐PrEP and LAI‐PrEP.

### 2.2 Data collection

During the PrEP15‐19 recruitment and follow‐up visits, a structured questionnaire was administered to a convenience sample of study participants between December 2020 and February 2022. Participants in the non‐PrEP arm were contacted via email and/or smartphone and were given the option to complete the questionnaire either in person or by phone. Participants recruited through RDS completed the questionnaire at enrolment. The questionnaire was administered in person by a research assistant trained in the online system. Interviewers had extensive experience in data collection for research involving adolescents from sexual and gender minority populations. The questionnaire comprised 17 questions assessing awareness and hypothetical intention to use ED‐PrEP and LAI‐PrEP. Following the awareness question on PrEP modalities, interviewers provided standardized explanations of the methods “Event‐driven HIV PrEP consists of taking two pills 2–24 hours before sexual intercourse, followed by two additional pills, one 24 hours later and another 48 hours later, to prevent HIV acquisition. This regimen is also referred to as 2+1+1”; and “LAI‐PrEP consists of receiving an injection in the arm or buttock every 2 months to prevent HIV acquisition.” At the time of data collection, ED‐PrEP was not yet included in Brazilian guidelines.

### 2.3 Variables

The outcome variables, awareness of ED‐PrEP and LAI‐PrEP, were assessed using the following questions: (1) “Before this study, had you heard about ED‐PrEP, a PrEP regimen indicated for MSM who plan their sexual encounters in advance or have sex less frequently?” (no, yes); and (2) “Before this study, had you heard about LAI‐PrEP, a form of PrEP administered via an injection in the arm or buttock that provides HIV protection for 2 months?” (no, yes).

The outcome variables, intention to use both PrEP modalities, were assessed using the following questions: (3) “Would you consider using ED‐PrEP for HIV prevention?” (no, yes); and (4) “Would you consider using LAI‐PrEP in any situation?” (no, yes).

Socio‐demographic data, experiences of discrimination and violence, and sexual behaviour characteristics were obtained from the socio‐behavioural questionnaire administered at enrolment in the PrEP15‐19 study. Information on prior daily oral PrEP use was collected from clinical follow‐up records and pill dispensing data. The explanatory variables included:

Socio‐demographic factors: study site (Salvador, São Paulo); age at interview (15–17 years or 18 and older); study population (AYMSM or AYTGW); race/skin colour (White, Black and Brown); education (elementary, high school or higher education). Socio‐economic status (high, middle or low) was classified using latent class analysis, as described by Magno et al. [16];

Sexual behaviour: number of casual sex partners in the past 3 months (fewer than three or none, three or more); engagement in sex work (no, yes); condom use frequency in receptive anal sex with a steady or casual partner (never/rarely, sometimes, always/often); and condom use frequency in insertive anal sex with a steady or casual partner (never/rarely, sometimes, always/often);

Discrimination and violence: lifetime discrimination based on sexual orientation or gender identity (no, yes); discrimination in healthcare settings (no, yes); and physical aggression due to sexual orientation or gender identity (no, yes);

Previous daily oral PrEP use: adherence was measured using the medication possession ratio (MPR), an indirect adherence measure. Participants were classified as having high adherence (MPR > 0.90), low/moderate adherence (MPR ≤ 0.90) based on a previously established cut‐off [17] or never used PrEP. PrEP discontinuation was categorized as completely discontinued, discontinued at least once or never discontinued. A participant was considered to have discontinued PrEP if they missed a scheduled appointment for 90 days or more.

Other variables describing anticipated barriers and facilitators to ED‐PrEP and LAI‐PrEP use were included for descriptive purposes only and were not incorporated into the modelling strategy. These were assessed through multiple‐choice questions.

### 2.4 Data analysis

We described the proportions of socio‐demographic characteristics, sexual behaviour, experiences of discrimination and violence, and previous daily oral PrEP use variables with 95% confidence intervals (95% CI), stratified by survey respondents (included) and non‐respondents (not included). Additionally, we reported awareness and intention to use ED‐PrEP and LAI‐PrEP, along with anticipated barriers and facilitators, stratified by previous experience with daily oral PrEP use, as well as the degree of difficulty associated with anticipated barriers to PrEP use. Participants with missing data were excluded from the analysis.

Pearson's chi‐square or Fisher's exact test was used for univariate associations (*p*‐value ≤ 0.05). Univariate and multivariate analyses were performed using binomial logistic regression to estimate adjusted odds ratios (OR) with 95% CI for associations between explanatory variables and outcomes. Model selection was guided by removing variables with *p*‐value > 0.2 from bivariate comparisons, and using the Akaike Information Criterion and the Bayesian Information Criterion to optimize model fit. The final model was refined for parsimony. Pearson's test assessed goodness‐of‐fit. All analyses were conducted in Stata 16.0 (StataCorp, 2019) and R (R CORE TEAM, 2022).

### 2.5 Ethical aspects

This study's protocol was approved by the ethical review committee (ERC) of the WHO and by the local ERCs of *Universidade de São Paulo* (protocol number 70798017.3.0000.0065), *Universidade Federal da Bahia* (protocol number 01691718.1.0000.5030) and *Universidade Federal de Minas Gerais* (protocol number 17750313.0.0000.5149). All adolescents aged 18 and 19 provided written informed consent. For those under 18, each city followed a distinct protocol based on local court decisions, requiring parental or guardian consent and/or adolescent assent, depending on jurisdiction.

## 3 RESULTS

A total of 1221 participants were enrolled in the cohort at this analysis, with 597 (48.9%) responding to the questionnaire on PrEP awareness and intention to use. Regarding previous experience with daily oral PrEP, the majority had low to moderate adherence (40.9%) and did not discontinue PrEP (45.1%) during the study period. Most participants were from São Paulo (58.7%), aged 18 or older (85.8%), identified as AYMSM (90.2%) and were Black or Brown (76.1%). The majority had medium or higher education (92.1%) and belonged to the middle socio‐economic level (57.8%). Regarding sexual behaviour, 54.9% reported having at least three casual sexual partners in the past 3 months, 3.1% reported engagement in sex work and approximately 60% reported always or often using a condom during receptive or insertive anal sex. In terms of discrimination and violence, 32.6% reported lifetime discrimination based on sexual orientation or gender identity, including 10.8% who experienced discrimination in health services. Reports of physical aggression were low (2.2%). Differences were identified between survey respondents and non‐respondents in the following characteristics: oral PrEP adherence and discontinuation, study site, socio‐economic level, engagement in sex work, frequency of condom use during insertive anal sex with steady or casual partners and experiences of physical aggression related to sexual orientation (Table 1).

**Table 1:** Characteristics of AYMSM and AYTGW stratified by survey completion status, PrEP15‐19, December 2020–February 2022

	Included (*n* = 597)		Not included (*n* = 624)		
Variables	*n*	%	*n*	%	*p*‐value*
**Adherence by medication possession ratio**					<0.001
High	187	32.6	314	50.4	
Low and moderate	235	40.9	194	31.1	
Never used PrEP	152	26.5	115	18.5	
**Discontinuation of PrEP**					<0.001
Discontinued	84	14.1	47	7.5	
Discontinued but used again	92	15.4	43	6.9	
Never discontinued	269	45.1	418	67.1	
Never used PrEP	152	25.5	115	18.5	
**Socio‐demographic**					
**Site**					0.015
Salvador	233	41.3	215	34.5	
São Paulo	331	58.7	409	65.5	
**Age**					0.899
15–17 years old	85	12.2	148	22.6	
18 years old and older	512	85.8	481	77.4	
**Study population**					0.080
AYMSM	469	90.2	542	86.9	
AYTWG	51	9.8	82	13.1	
**Race/colour**					0.337
White	124	23.9	191	30.6	
Black and Brown	395	76.1	433	69.4	
**Education**					0.067
Elementary school	47	7.9	62	10.0	
High school	424	71.5	405	65.3	
Higher education	122	20.6	153	24.7	
**Socio‐economic level**					<0.001
High	90	19.2	281	45.0	
Middle	271	57.8	175	28.1	
Low	108	23.0	168	26.9	
**Sexual behaviour**					
**Number of casual sex partners in the previous 3 months**					<0.001
Less than three or none	269	45.1	180	28.9	
Three or more	328	54.9	444	71.1	
**Engagement in sex work**					<0.001
No	553	96.9	525	91.0	
Yes	18	3.1	52	9.0	
**Frequency of condom use in receptive anal sex with a steady or casual partner**					0.914
Never/rarely	86	17.9	95	18.9	
Sometimes	106	22.1	108	21.5	
Often/always	288	60.0			
**Frequency of condom use in insertive anal sex with a steady or casual partner**					0.017
Never/rarely	33	9.7	61	16.5	
Sometimes	104	30.4	115	31.1	
Frequently/always	205	59.9	194	52.4	
**Discrimination and violence**					
**Discrimination related to sexual orientation and gender identity**					0.080
No	250	67.4	375	72.8	
Yes	121	32.6	140	27.2	
**Discrimination in health service**					0.144
No	346	89.2	516	86.0	
Yes	42	10.8	84	14.0	
**Physical aggression related to sexual orientation**					<0.001
No	577	97.8	578	92.8	
Yes	13	2.2	45	2.2	

Abbreviations: AYMSM, adolescents and young men who have sex with men; AYTGW, adolescents and young transgender women; ED‐PrEP, event‐driven pre‐exposure prophylaxis; LAI‐PrEP, long‐acting injectable pre‐exposure prophylaxis; PrEP, pre‐exposure prophylaxis.

^*^ Pearson's chi‐square test.

For ED‐PrEP, most participants had no prior awareness (84.7%). After learning about the modality, 56.4% expressed intention to use it, significantly higher among those who never used daily oral PrEP (72.2%) compared to those who previously or currently used it (51.2%) (*p* < 0.001). Most participants (77.1%) stated they would not switch from daily PrEP to ED‐PrEP if it became available in the *SUS* despite both regimens using tenofovir disoproxil fumarate and entricitabine (TDF/FTC). Regarding potential barriers, 31.7% reported a high likelihood of engaging in unplanned sexual intercourse and not having enough time to initiate ED‐PrEP before sex. For LAI‐PrEP, most participants had no prior awareness (82.0%). After learning about it, 81.5% reported intention to use it. Additionally, intention to use was higher among those who previously or currently used daily oral PrEP (84.0%) compared to those who never used it (73.6%) (*p* < 0.01) (Table 2).

**Table 2:** Awareness and intention to use ED‐PrEP and LAI‐PrEP among AYMSM and AYTGW stratified by previous daily oral PrEP use, PrEP15‐19, December 2020–February 2022

	Total (*N* = 597)		No previous daily oral PrEP use (*n* = 152)		Previous daily oral PrEP use (*n* = 445)		
Variables	*N*	%	*n*	%	*n*	%	*p*‐value*
**ED‐PrEP**							
**Awareness**							0.073
No	503	84.7	133	89.3	370	83.1	
Yes	91	15.3	16	10.7	75	16.9	
**Intention to use ED‐PrEP**							<0.001
No	256	43.6	40	27.8	216	48.8	
Yes	331	56.4	104	72.2	227	51.2	
**Would change regimen if available in *SUS***							0.124
No	320	77.1	124	81.6	336	75.5	
Yes	137	22.9	28	18.4	109	24.5	
**Possibility of having an unplanned intercourse and not having time to use it**							0.627
High	175	31.7	34	28.6	141	32.6	
Medium	156	28.3	33	27.7	123	28.4	
Low	151	27.3	38	31.9	113	26.1	
None	70	12.7	14	11.8	56	13.0	
**LAI‐PrEP**							
**Awareness**							<0.001
No	486	82.0	136	91.9	350	78.7	
Yes	107	18.0	12	8.1	95	21.3	
**Intention to use LAI‐PrEP**							0.005
No	109	18.5	38	26.4	71	16.0	
Yes	479	81.5	106	73.6	373	84.0	

Abbreviations: AYMSM, adolescents and young men who have sex with men; AYTGW, adolescents and young transgender women; ED‐PrEP, event‐driven pre‐exposure prophylaxis; LAI‐PrEP, long‐acting injectable pre‐exposure prophylaxis; PrEP, pre‐exposure prophylaxis; SUS, Brazilian National Health System.

^*^Pearson's chi‐square test.

The main anticipated barriers to ED‐PrEP use were the need to plan sexual intercourse (46.7%) and the challenge of remembering the 2+1+1 regimen correctly (44.4%). For LAI‐PrEP, the most anticipated facilitators were perceived advantages over daily medication (33.2%), reduced concern about forgetting pills (21.4%) and not having to worry about HIV for 2 months (16.9%). The concern about forgetting pills was more prevalent among those who previously used daily oral PrEP (24.3%) compared to those who never used it (10.5%). The main anticipated barriers to LAI‐PrEP use were fear of injections (22.3%), inability to interrupt use before the next injection in case of side effects (16.8%), fear of needles (13.0%) and fear of pain from the injection (6.4%). Concern about being unable to stop use before the next injection was more frequent among those who previously used daily oral PrEP (18.9%) than among those who never used it (8.6%) (Table 3).

**Table 3:** Anticipated barriers and facilitators for intention to use ED‐PrEP and LAI‐PrEP among AYMSM and AYTGW, PrEP15‐19 study, Brazil, December 2020–February 2022

Variables	Total (*N* = 597)		No previous daily oral PrEP use (*n* = 152)		Previous daily oral PrEP use (*n* = 445)	
	*n*	%	*n*	%	*n*	%
**ED‐PrEP**						
**Anticipated barriers (*n* = 593)**						
Sexual intercourse planning	279	46.7	58	38.2	221	49.7
Correctly remember the 2+1+1 regimen	265	44.4	64	42.1	201	45.2
Not confident in the safety of PrEP	12	2.0	2	1.3	10	2.2
Others	25	4.2	7	4.6	18	4.0
None of the options	123	20.6	37	24.3	86	19.3
**LAI‐PrEP**						
**Anticipated barriers (*n* = 593)**						
Fear of injections	129	22.3	47	30.9	82	18.4
The impossibility of interrupting use before the next injection in case of side effects	97	16.8	13	8.6	84	18.9
Fear of needles	75	13.0	19	12.5	56	12.6
Fear of feeling the pain caused by injectable medication	37	6.4	2	1.3	35	7.9
It is new and does not have enough information	35	6.0	2	1.3	33	7.4
I will have to go to the service more often	31	5.4	4	2.6	27	6.1
I think the adverse events can be more dangerous	30	5.2	5	3.3	25	5.6
I do not think it is the safest method to prevent HIV	6	1.0	1	0.7	5	1.1
It is less discreet, because I have to expose myself more	6	1.0	3	2.0	3	0.7
None of the options	284	49.1	63	41.4	221	49.7
Others	30	5.2	15	9.9	15	3.4
**Anticipated facilitators (*n* = 257)**						
Better than taking daily medication	192	33.2	42	27.6	150	33.7
Not having to worry about forgetting to take my pills	124	21.4	16	10.5	108	24.3
Not having to worry about HIV for 2 months	98	16.9	50	32.9	48	10.8
It is better than taking medicine every time you have sex	84	14.5	13	8.6	71	16.0
Not having to worry about carrying the medication with you	77	13.3	15	9.9	62	13.9
No one will see the PrEP pills	22	3.8	7	4.6	15	3.4
Fewer side effects	15	2.6			13	2.9
Others	2	0.3	0	0.0	2	0.4

Abbreviations: AYMSM, adolescents and young men who have sex with men; AYTGW, adolescents and young transgender women; ED‐PrEP, event‐driven pre‐exposure prophylaxis; HIV, human immunodeficiency virus; LAI‐PrEP, long‐acting injectable pre‐exposure prophylaxis; PrEP, pre‐exposure prophylaxis.

Most participants did not anticipate difficulty with LAI‐PrEP in the following scenarios: attending the clinic every 2 months for injections (76.3%), concerns about pain from the injection (63.8%), switching to daily PrEP for 12 months if injectable PrEP had to be discontinued (50.5%) and discussing this PrEP modality with their partner (84.1%) (Table 4).

**Table 4:** Difficulty degree of anticipated barriers to PrEP use, PrEP15‐19 study, Brazil, December 2020–February 2022

Variables (*N* = 597)	*n*	%
**Going to the service every 2 months to take PrEP**		
No difficulty	421	76.3
Slight difficulty	78	14.1
Moderate difficulty	43	7.8
Great difficulty	10	1.8
**Fear of feeling pain caused by the injectable medication**		
No difficulty	352	63.8
Slight difficulty	84	15.2
Moderate difficulty	67	12.1
Great difficulty	49	8.9
**Taking daily PrEP for 12 months if you have to discontinue injectable PrEP**		
No difficulty	279	50.5
Slight difficulty	87	15.8
Moderate difficulty	101	18.3
Great difficulty	85	15.4
**Discussing this PrEP modality with their partner**		
No difficulty	464	84.1
Slight difficulty	47	8.5
Moderate difficulty	27	4.9%
Great difficulty	14	2.5

Abbreviation: PrEP, pre‐exposure prophylaxis.

In multivariate analysis, participants with low and moderate adherence to daily oral PrEP (OR = 1.79; 95% CI: 1.04–3.08) were more likely to intend to use ED‐PrEP, whereas those who always or often used condoms in insertive anal sex with steady or casual partners (OR = 0.37; 95% CI: 0.15–0.90) were less likely to intend to use it. For LAI‐PrEP, intention to use was higher among participants with middle (OR = 1.93; 95% CI: 1.05–3.53) or low socio‐economic status (OR = 3.13; 95% CI: 1.30–7.51) and among those who had three or more casual partners in the past 3 months (OR = 2.25; 95% CI: 1.30–3.89). Participants who had not previously used daily oral PrEP were less likely to intend to use LAI‐PrEP compared to those who previously used and never discontinued daily oral PrEP (OR = 0.31; 95% CI: 0.11–0.92) (Table 5).

**Table 5:** Factors associated with intention to use ED‐PrEP and LAI‐PrEP in AYMSM and AYTGW, December 2020–February 2022 (*N* = 597)

Variables	Intention to use ED‐PrEP				Intention to use LAI‐ PrEP			
	Univariate			Multivariate	Univariate			Multivariate
	%	*p*‐value	OR (95% CI)	aOR (95% CI)	%	*p*‐value	OR (95% CI)	aOR (95% CI)
**Socio‐demographic**								
**Site**		0.001^*^				0.102^*^		
Salvador	46.78				84.98			
São Paulo	61.18		1.79 (1.27–2.52)	1.17 (0.69–1.99)	79.57		0.69 (0.44–1.08)	0.85 (0.49–1.47)
**Age**		0.267^*^				0.396^*^		
15–17 years old	60.61				78.95			
18 years and older	55.16		0.80 (0.54–1.19)		82.2		1.23 (0.76–1.99)	
**Study population**		0.923^*^				0.716^*^		
AYMSM	53.65				82.44			
AYTWG	52.94		0.97 (0.54–1.73)		80.39		0.87 (0.42–1.81)	
**Race/colour**		0.746^*^				0.401^*^		
White	52.42				79.67			
Black and Brown	54.08		1.07 (0.71–1.60)		82.99		1.24 (0.75–2.08)	
**Education**		0.484^*^				0.220^*^		
Elementary school	63.04				72.34			
High school	56.73		0.77 (0.41–1.44)		82.69		1.83 (0.92–3.63)	
Higher education	52.89		0.66 (0.33–1.32)		80.99		1.62 (0.74–3.57)	
**Socio‐economic level**		0.173^*^				0.043^*^		
High	56.48				76.85			
Middle	52.04		0.84 (0.53–1.31)	0.87 (0.44–1.71)	84.07		1.59 (0.91–2.77)	1.93 (1.05–3.53)
Low	43.33		0.59 (0.33–1.04)	0.65 (0.28–1.49)	90		2.71 (1.19–6.16)	3.13 (1.30–7.51)
**Previous daily oral PrEP use**								
**Discontinuation of PrEP**		< 0.001^*^				0.013^*^		
Never discontinued	48.13				82.53			
Discontinued but used again	50		1.08 (0.67–1.73)		90.22		1.95 (0.92–4.16)	1.64 (0.75–3.59)
Discontinued	62.65		1.81 (1.09–3.00)		81.93		0.96 (0.51–1.82)	4.06 (0.51–2.23)
Never used	72.22		2.80 (1.81–4.33)		73.61		0.59 (0.36–0.96)	0.31 (0.11–0.92)
**Adherence by medication possession ratio**		< 0.001^*^				0.011^*^		
High	44.26				83.4			
Low and moderate	56.22		1.62 (1.10–2.38)	1.79 (1.04–3.08)	86.02		1.22 (0.71–2.10)	
Never used	72.22		3.28 (2.10–5.12)	2.30 (0.48–11.05)	73.61		0.56 (0.33–0.92)	
**Sexual behaviour**								
**Engagement in sex work**		0.199^*^				<0.001^†^		
No	56.86				82.82			
Yes	41.18		0.53 (0.20–1.42)		44.44		0.17 (0.06–0.43)	
**Number of casual sex partners in the previous 3 months**		0.667^*^				0.064^*^		
Less than three or none	57.36				78.2			
Three or more	55.59		0.93 (0.67–1.29)		84.16		1.48 (0.98–2.50)	2.25 (1.30–3.89)
**Frequency of condom use in receptive anal sex with a steady or casual partner**		0.827^*^				0.512^*^		
Never/rarely	54.12				85.71			
Sometimes	51.43		0.90 (0.51–1.59)		85.85		1.01 (0.45–2.29)	
Always/often	54.93		1.03 (0.64–1.68)		81.75		0.75 (0.38–1.48)	
**Frequency of condom use in insertive anal sex with a steady or casual partner**		0.150^*^				0.338^*^		
Never/rarely	65.63				75.76			
Sometimes	57.84		0.72 (0.31–1.65)	0.58 (0.23–1.51)	86.54		2.06 (0.78–5.45)	
Always/often	49.75		0.52 (0.24–1.13)	0.37 (0.15–0.90)	84.08		1.69 (0.70–4.08)	
**Discrimination and violence**								
**Discrimination related to sexual orientation**		0.937^*^				0.397^*^		
No	49.2				86			
Yes	48.76		0.98 (0.63–1.52)		82.64		0.76 (0.43–1.40)	
**Discrimination in health services**		0.250^*^				0.859^*^		
No	49.42				84.39			
Yes	47.62		0.93 (0.49–1.77)		83.33		0.92 (0.39–2.19)	
**Physical aggression related to sexual orientation**		0.859^*^				0.559^*^		
No	56.32				81.47			
Yes	53.85		0.90 (0.30–2.73)		84.62		1.25 (0.27–5.73)	
**Pearson's GOF test (*p*‐value)**				0.973				0.913

Abbreviations: aOR, adjusted odds ratio; AYMSM, adolescents and young men who have sex with men; AYTGW, adolescents and young transgender women; ED‐PrEP, event‐driven pre‐exposure prophylaxis; GOF, goodness‐of‐fit; LAI‐PrEP, long‐acting injectable pre‐exposure prophylaxis; OR, odds ratio; PrEP, pre‐exposure prophylaxis.

^*^ Pearson's chi‐square.

^†^ Fisher's exact test.

## 4 DISCUSSION

Our results showed that awareness of both PrEP modalities remains low among adolescents and young people in two highly populated urban centres in Brazil, regardless of previous daily oral PrEP use. However, after receiving information, more than two‐thirds of those with no prior experience with daily oral PrEP expressed an intention to use ED‐PrEP or LAI‐PrEP.

Preference and intention to use different PrEP modalities vary across studies, influenced by differences in study populations, outcome measures and prior experience with injectable PrEP, making direct comparisons challenging [18, 19, 20, 21, 22]. In our study, the intention to use LAI‐PrEP was higher than reported in studies with adults [18, 19, 20]. Previous studies reported LAI‐PrEP preference ranging from 44% to 67% among adult MSM in the United States, France and Brazil [18, 19, 23].

Studies assessing LAI‐PrEP preference among current users suggest that acceptability increases with experience. In the HPTN 077 cohort, baseline preference for LAI‐PrEP with cabotegravir was 56% among U.S. participants and 67% among non‐U.S. participants assigned male at birth, increasing to 68% and 93%, respectively, after the final injection [20]. Similarly, the ECLAIR Trial reported that 74% preferred LAI‐PrEP with cabotegravir over oral formulations, and 79% were willing to continue use [21].

Data comparing ED‐PrEP preference with other PrEP modalities among MSM vary. A study conducted in Amsterdam (2015–2016) found that ED‐PrEP was less preferred (27%) compared to daily oral PrEP (73%). In that study, older MSM (≥ 45 years) with a steady partner and fewer instances of condomless anal sex were more likely to choose ED‐PrEP [22].

Our data aligns with previous qualitative findings from the PrEP15‐19 study, which indicated that AYMSM and AYTGW demonstrated interest in LAI‐PrEP, primarily due to its ability to eliminate the burden of daily oral PrEP use and reduce concerns about forgetting medication. Participants also identified reduced dosing frequency, ease of use and increased confidentiality from family members as key advantages of injectable PrEP. However, fear of injections, reliance on healthcare professionals for administration, concerns about effectiveness and potential adverse effects were reported as barriers to LAI‐PrEP use [24].

Participants who reported consistent condom use in insertive anal sex with steady or casual partners were less likely to intend to use ED‐PrEP. This finding suggests that individuals who favour non‐medication‐based prevention strategies may be less inclined to adopt new PrEP regimens, particularly those that require event‐driven management for each sexual encounter. Additionally, daily or long‐acting PrEP regimens may be more effective in providing consistent protection across both casual and steady partners. Sexual practices are dynamic and may evolve based on relationship context [25]. In our study, 42.7% and 89.6% of participants who reported insertive anal sex also engaged in receptive anal sex often or sometimes, respectively. Preferences for HIV prevention modalities may also be influenced by sexual practices. For example, a study in France found that MSM who engaged in condomless anal sex were more likely to be willing or very willing to use daily oral PrEP, ED‐PrEP and LAI‐PrEP [19].

Low adherence to daily oral PrEP was associated with a greater likelihood of intending to use ED‐PrEP. One possible explanation is that daily PrEP requires consistent effort to remember medication use, which may be particularly challenging for individuals with variable or infrequent sexual activity. Additionally, daily use can lead to pill fatigue [26], especially among those with prior experience with daily oral PrEP and low adherence. In this context, ED‐PrEP offers a viable alternative for individuals who wish to discontinue daily use while maintaining HIV protection when needed [10].

Thus, ED‐PrEP has the potential to expand PrEP coverage for individuals with less frequent or fluctuating sexual activity over time [10]. This may explain the lower intention to use ED‐PrEP observed in our study, as the profile of these adolescents may align more closely with daily oral PrEP or injectable PrEP use. Furthermore, greater dissemination of information about the ED‐PrEP 2+1+1 protocol is needed. Increasing awareness and education on ED‐PrEP could help address current gaps in knowledge and uptake.

The intention to use LAI‐PrEP was high, particularly among individuals who had previously used daily oral PrEP, compared to those who had never used it. This finding underscores the potential for significant regimen switching as LAI‐PrEP becomes available, emphasizing the need to integrate structured support mechanisms for PrEP‐experienced users. Facilitating a smooth transition between PrEP modalities may help sustain engagement in HIV prevention, as more convenient options can mitigate pill fatigue [26] and reduce adherence‐related stress [27], ultimately enhancing long‐term PrEP persistence.

In Brazil, LAI‐PrEP with cabotegravir is currently implemented only in select implementation studies (i.e. PrEP15‐19 Choices and ImPrEP CAB Brasil) [1, 28, 29]. Initial findings from the PrEP15‐19 Choices study suggest that adolescents from more vulnerable backgrounds—specifically Black adolescents, those with lower educational attainment and younger individuals aged 15–17 years—are more likely to select injectable cabotegravir over oral formulations as their initial PrEP choice [30].

Given that daily pill use remains a challenge for adolescents and young people often due to forgetfulness and the burden of maintaining a daily regimen providing tailored counselling and programmatic support for product switching will be essential for optimizing PrEP delivery and expanding coverage among AYMSM and AYTGW globally. Furthermore, it is crucial to emphasize the importance of choice in PrEP delivery, particularly with the potential introduction of new long‐acting injectable (LAI‐PrEP) options, such as lenacapavir, which is administered as a subcutaneous injection every 26 weeks [31]. Expanding PrEP options to include longer‐acting formulations could further enhance adherence, reduce stigma and increase acceptability among key populations at high risk of HIV acquisition.

The socio‐economic status of adolescents in this study directly influenced the intention to use LAI‐PrEP, with those from middle‐ and low‐income backgrounds being more likely to prefer this modality. These findings align with previous studies conducted among low‐ and middle‐income adult MSM in the United States [18, 32]. Long‐acting PrEP may be particularly advantageous for individuals with lower socio‐economic status, especially adolescents, due to structural barriers such as unstable housing, the need to balance work and school responsibilities, and heightened vulnerability to stigma and discrimination.

Additionally, having a higher number of sexual partners in the 3 months prior to the survey was significantly associated with a greater intention to use LAI‐PrEP among adolescents in this study. This reinforces the assumption that long‐acting PrEP modalities may have high acceptability among individuals at increased risk of HIV acquisition, particularly those seeking greater convenience and protection in HIV prevention, a trend also observed in other studies [18, 33, 34]. One possible explanation for the preference for LAI‐PrEP among adolescents with multiple partners is the perception of prolonged security, combined with facilitated adherence, as it requires only returning for scheduled injections rather than daily pill‐taking. This aligns with previous research demonstrating that individuals with higher sexual activity levels may gravitate towards PrEP modalities that minimize adherence challenges while ensuring continuous protection against HIV [18, 24, 34].

This study has some limitations. The cross‐sectional design does not allow for causal inference, and the convenience sampling approach limits the generalizability of findings. This acceptability study was conducted using a questionnaire administered to a sub‐sample of participants from the PrEP15‐19 project. While certain characteristics did not significantly differ between participants included and not included in this sub‐study. Voluntary participation may have introduced response bias, limiting generalizability. Additionally, the network recruitment strategy was more extensively implemented at the São Paulo site, leading to a greater proportion of participants who had never used PrEP before. To mitigate potential selection bias and account for site‐specific differences, we included the study site as an adjustment variable in the analysis. Moreover, this study assessed hypothetical intention to use ED‐PrEP and LAI‐PrEP, rather than real‐world choice, as these PrEP products were not yet available at the time of data collection. This could have been influenced by limited community awareness and lack of peer engagement, affecting participant responses. Additionally, the highly reported intention to use PrEP may be affected by participation bias, as individuals already engaged in HIV prevention initiatives may have a greater predisposition to express interest in new prevention modalities. Despite these limitations, our findings align with existing literature and provide important insights for developing tailored HIV prevention strategies for populations most vulnerable to HIV acquisition.

## 5 CONCLUSIONS

Awareness of ED‐PrEP and LAI‐PrEP is low among key population adolescents in this study, highlighting the need to expand discussions on new HIV prevention strategies, particularly for those with limited access. Beyond introducing alternative PrEP options, clearer information on ED‐PrEP is necessary to ensure correct usage, enabling informed choice for individuals who can plan sexual activity in advance.

After being introduced to ED‐PrEP and LAI‐PrEP, participants expressed high intention to use both methods, with greater preference for LAI‐PrEP. Since PrEP choice depends on prior HIV prevention experiences and sexual behaviours, multiple options must be available for adolescents to select based on their needs and preferences. New HIV prevention methods should be incorporated into counselling in prevention services, ensuring adolescents understand available options, indications and risks to support informed preventive decisions.

## COMPETING INTERESTS

The authors declare that they have no competing interests.

## AUTHORS’ CONTRIBUTIONS

LM coordinated data analysis, interpreted findings, contributed to manuscript drafting and provided critical revisions. BOL acquired cohort data, assisted in data analysis and interpretation, conducted literature searches and contributed to manuscript drafting. FS acquired cohort data, assisted in data interpretation and provided critical manuscript revisions. LD conducted literature searches, contributed to data interpretation and provided critical revisions. ID, AG and LM designed and conceptualized the PrEP15‐19 study, secured funding, coordinated data collection and contributed to manuscript drafting. All authors reviewed and approved the final version of the article.

## FUNDING

This work was supported by UNITAID, which funded the PrEP15‐19 study, and by the Brazilian National Health System (SUS), which provided PrEP medications, condoms, rapid tests and additional care services for PrEP15‐19 participants. UNITAID accelerates access to innovative health products and lays the foundations for their scale‐up by countries and partners, and it is a hosted partnership of WHO. These services were delivered through the Department of Chronic Diseases and Sexually Transmitted Infections of the Ministry of Health, the Bahia State and Salvador City Departments of Health, the São Paulo State and City Departments of Health, and the São Paulo AIDS Program. The study sponsors had no role in study design, data collection, analysis or interpretation, manuscript writing, or the decision to submit the paper for publication.

## Data Availability

The informed consent process ensured that participants’ confidentiality and anonymity were strictly protected, and that only PrEP15‐19 study investigators could access the collected data. Ethical approval for the study was obtained from the Research Ethics Review Committees of the Universidade de São Paulo (protocol number 70798017.3.0000.0065), Universidade Federal da Bahia (protocol number 01691718.1.0000.5030) and Universidade Federal de Minas Gerais (protocol number 17750313.0.0000.5149). To ensure participant confidentiality and anonymity, researchers may contact the Research Ethics Committee of Universidade de São Paulo (Comissão de Ética para Análise de Projetos de Pesquisa, email: cappesq.adm@hc.fm.usp.br) for requests related to data access for analyses conducted in this manuscript. Due to the sensitive nature of the data, public sharing is not permitted.
